# Determination of gamma camera calibration factors for quantitation of therapeutic radioisotopes

**DOI:** 10.1186/s40658-018-0208-9

**Published:** 2018-05-02

**Authors:** Wei Zhao, Pedro L. Esquinas, Xinchi Hou, Carlos F. Uribe, Marjorie Gonzalez, Jean-Mathieu Beauregard, Yuni K. Dewaraja, Anna Celler

**Affiliations:** 1 Department of Physics and Astronomy, University of British Columbia, Vancouver, BC Canada; 2 Medical Imaging Research Group, Department of Radiology, University of British Columbia, Vancouver, BC Canada; 3 Department of Molecular Oncology, BC Cancer Research Center, Vancouver, BC Canada; 4 Vancouver Coastal Health Authority, Vancouver, BC Canada; 5 Department of Medical Imaging, CHU de Quebec-Université Laval, Quebec City, QC Canada; 6 Department of Radiology and Nuclear Medicine, Université Laval, Quebec City, QC Canada; 7 Department of Radiology, University of Michigan Medical School, Ann Arbor, MI USA

**Keywords:** Gamma camera calibration, TEW, Quantification, Iodine-131, Lutetium-177, Rhenium-188

## Abstract

**Background:**

Camera calibration, which translates reconstructed count map into absolute activity map, is a prerequisite procedure for quantitative SPECT imaging. Both planar and tomographic scans using different phantom geometries have been proposed for the determination of the camera calibration factor (CF). However, there is no consensus on which approach is the best. The aim of this study is to evaluate all these calibration methods, compare their performance, and propose a practical and accurate calibration method for SPECT quantitation of therapeutic radioisotopes. Twenty-one phantom experiments (Siemens Symbia SPECT/CT) and 12 Monte Carlo simulations (GATE v6.1) using three therapy isotopes (^131^I, ^177^Lu, and ^188^Re) have been performed. The following phantom geometries were used: (1) planar scans of point source in air (PS), (2) tomographic scans of insert(s) filled with activity placed in non-radioactive water (HS + CB), (3) tomographic scans of hot insert(s) in radioactive water (HS + WB), and (4) tomographic scans of cylinders uniformly filled with activity (HC). Tomographic data were reconstructed using OSEM with CT-based attenuation correction and triple energy window (TEW) scatter correction, and CF was determined using total counts in the reconstructed image, while for planar scans, the photopeak counts, corrected for scatter and background with TEW, were used. Additionally, for simulated data, CF obtained from primary photons only was analyzed.

**Results:**

For phantom experiments, CF obtained from PS and HS + WB agreed to within 6% (below 3% if experiments performed on the same day are considered). However, CF from HS + CB exceeded those from PS by 4–12%. Similar trend was found in simulation studies. Analysis of CFs from primary photons helped us to understand this discrepancy. It was due to underestimation of scatter by the TEW method, further enhanced by attenuation correction. This effect becomes less important when the source is distributed over the entire phantom volume (HS + WB and HC).

**Conclusions:**

Camera CF could be determined using planar scans of a point source, provided that the scatter and background contributions are removed, for example using the clinically available TEW method. This approach is simple and yet provides CF with sufficient accuracy (~ 5%) to be used in clinics for radiotracer quantification.

## Background

In single-photon emission computed tomography (SPECT), quantification of radiotracer distribution has recently become an increasingly important component of many clinical studies [1, 2]. In particular, quantitative SPECT can be very helpful in the diagnosis of multi-vessel heart disease and assessment of myocardial blood flow reserve [3], as well as in quantitative evaluation of the lungs, kidneys, brain [4] and other organs.

However, the most important role activity quantitation has to play is in the targeted radionuclide therapies (TRT) [5]. The assessment of tumor burden, prediction of potentially critical organs and normal tissue toxicities, and calculation of the radiation dose are all necessary elements of the personalized, image-based therapy planning as well as evaluation of patient’s response to this therapy. They all require accurate absolute quantification of the amount of the radioactive material that is localized in tumor(s) and critical organs and characterization of its changes over time (biokinetics) [6, 7].

There are three essential steps, which have to be performed for quantification of SPECT images. The first step involves quantitative SPECT reconstructions. Since the data acquired in projections are affected by physical phenomena such as photon attenuation and scatter, collimator blurring, camera dead-time and partial volume effects; in order to get quantitatively accurate images, all these factors must be properly compensated for during the reconstruction process. Fortunately, in the past few decades, considerable technical advancement has been achieved in both SPECT hardware and data processing software. Particularly, with the introduction of hybrid SPECT/CT imaging systems and the development of statistical iterative reconstruction algorithms, quantitative reconstructions have become available for the majority of the commercial SPECT/CT cameras [8–10].

The second step is to apply camera calibration factor (CF) to the reconstructed images, which will translate the three-dimensional (3D) count maps into 3D activity maps. It is important to stress at this point that CF provides only a numerical coefficient necessary for this “translation”. The value of CF depends on the energy of the measured photons; therefore, it is radioisotope specific and represents the joint sensitivity of the camera and the collimator for detection of a particular isotope’s emissions in the energy window(s) that is used for data acquisition. Please note that the value of CF might be influenced by the potential errors in dose calibrator readings when measuring the activity.

Finally, in order to obtain a quantitative value of the activity contained in any particular volume of tissue (for example in an organ or a tumor), the third step involving segmentation of this activity map must be performed. As segmented volumes will be affected by partial volume effects (PVE), for accurate activity quantification, appropriate PVE correction methods must be applied [11]. For example, one such method would be to use experimentally determined recovery coefficients (RC) [12, 13].

The most reliable method to determine CF of the camera is to perform an experimental measurement using an accurately calibrated radioactive source. Considering that quantitative reconstruction methods generate images from primary photons (PP) (as quantitative reconstruction has already removed the scattered photons and corrected for losses due to attenuation), CF must relate these PP images to the activity which produced them.

Different camera calibration methods have been proposed, but there is still no consensus which method is the best. Some researchers use planar scans of a small (point-like) source (PS) placed in air at a certain distance (usually 20–30 cm) from the collimator surface [14–19]. This is a simple method where the CF is directly calculated from the acquired planar images. Care must be taken, however, that photon scatter is accounted for and that attenuation in the source and source support are minimized. Different small-volume geometries ranging from a vial to a syringe [15, 18]and a small container [14] to a petri dish (following NEMA protocol for camera sensitivity test [19]) have been employed. Some researchers even performed tomographic scans of such a point source [16]; however, it is not clear what would be the advantage of such acquisition.

Alternatively, tomographic scans of large cylindrical phantoms containing accurately measured amounts of radioactive materials have been proposed [12, 13, 20–26]. This approach is more cumbersome, especially when radioisotopes with long half-lives are used. However, its rationale is that the geometry of the extended calibration phantom better models the body of a patient and the physical effects (photon attenuation and scatter) which occur in patients’ acquisitions. Therefore, all approximations (and potential inaccuracies) due to the clinical reconstruction method which may affect the accuracy of patient images will be replicated in the reconstructed images of the calibration phantom. The geometries which have been used in the extended phantom experiments can be divided into three categories: (a) small container(s) filled with activity (hot sources (HS)) placed in the large cylinder filled with non-radioactive water (cold background (CB)) [13, 22, 23, 26], (b) small container(s) with activity placed in the large cylinder filled with radioactive water (warm background (WB)) [21], and (c) large cylinders with no inserts, filled uniformly with activity (hot-cylinder (HC)) [12].

The purpose of the present study is to evaluate all these methods, compare their performance and check if, and under what conditions, the planar calibration and tomographic calibration produce equivalent results. A large series of phantom experiments, as well as extensive simulation studies, have been performed. The objective of the simulations (done with GATE Monte Carlo program [27]) was to generate the true CF values, and to investigate and understand the physical effects, which may be responsible for the discrepancies observed between CFs obtained using different experimental methods. Three popular therapeutic radioisotopes (emitting beta particles and also gamma radiation) were investigated, namely, ^131^I, ^177^Lu and ^188^Re.

## Methods

Our study was composed of two parts: (1) phantom experiments and (2) Monte Carlo simulations. In both parts, ^131^I, ^177^Lu and ^188^Re radioisotopes were used, and in total, 21 experimental scans and 12 simulation runs were performed. The information about the isotopes’ half-lives, their most intensive gamma emissions and maximum and mean energy of their beta emission, is provided in Table 1.

**Table 1:** Decay characteristics of ^131^I [34], ^177^Lu [35], and ^188^Re [36]

Isotope	Half-life	Strongest *γ* emissions *E*_**γ**_ [keV] (*I*_*γ*_ [%])^a^	Mean *β* energy *E*_mean_ [keV]	Max *β* energy *E*_max_ [keV]
^131^I	8.03 days	284 (6.1)	181.9	970.8
		364 (81.5)		
		637 (7.2)		
		723 (1.8)		
^177^Lu	6.65 days	113 (6.2)	134.2	498.3
		208 (10.4)		
^188^Re	17.00 h	155 (15.6)	763	2120.4
		478 (1.1)		
		633 (1.4)		

^a^Only gammas with intensities higher than 1% were listed

### Phantom experiments

**Fig. 1:**
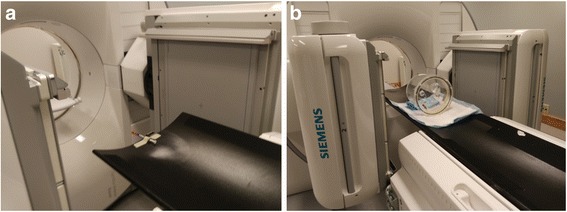
Examples of experimental configurations used in planar (**a**) and tomographic (**b**) acquisitions

For each isotope, the data were acquired using the following three experimental configurations (see Fig. 1 and Table 2):

A. Planar acquisition of a small source suspended in air (PS; Table 2: experiments #1, #6–7, and #15–17)
B. Tomographic (SPECT/CT) acquisition of hot inserts (spheres and/or cylinders) placed in non-radioactive water (HS + CB; Table 2: experiments #2–3, #8–9, and #18–19)
C. Tomographic (SPECT/CT) acquisition of the same set of hot inserts placed in radioactive water (HS + WB; Table 2: experiments #4–5, #10–13, and #20–21)

**Table 2:** Parameters of acquisitions and source activities used in phantom experiments

Experiment number	Isotope	Camera and collimator	Number of projections	Experimental configuration	Total phantom activity [MBq]	Source-collimator distance [cm]
1	^131^I	Symbia T and HE	1	A ➔ PS	24.35	25
2			60	B ➔ HS + CB	16.02	Non-circular orbit
3					20.76	
4				C ➔ HS + WB	89.54	
5					203.86	
6	^177^Lu	Symbia T and ME	1	A ➔ PS	11.70	36
7					13.10	35
8			90	B ➔ HS + CB	446.79	Non-circular orbit
9					277.50	
10				C ➔ HS + WB	681.26	
11					489.08	
12					2486.60	
13					2459.89	
14			96	D ➔ HC	659.60	
15	^188^Re	Symbia T and HE	1	A ➔ PS	14.15	30
16					16.25	13
17					119.02	13
18			90	B ➔ HS + CB	664.0	Non-circular orbit
19					554.0	
20				C ➔ HS + WB	491.0	
21					1193.0	

Additionally, for ^177^Lu, the following fourth configuration was used:

D. Tomographic (SPECT/CT) acquisition of a cylindrical phantom filled with uniform activity (HC; Table 2: experiment #14)

All data acquisitions were performed using Symbia SPECT/CT cameras (Siemens Healthineers, Germany). The acquisitions #6–13 for ^177^Lu and #15–21 for ^188^Re were performed at the Vancouver General Hospital, Nuclear Medicine Department, Vancouver (Canada). Experiments with ^131^I (acquisitions #1–5) were done at the Department of Radiology, University of Michigan Medical School, Ann Arbor (USA). And finally, the ^177^Lu acquisition #14 was performed at the Department of Radiology and Nuclear Medicine, Université Laval, Quebec (Canada). The acquisition conditions, the camera model, the collimators, and the total activities used in the experiments are specified in Table 2.

For experiments performed using configuration A, the volume of the point source was always equal to or less than 1 mL. In each case, a syringe containing the point source was suspended in air between the detectors and it was equally spaced from each collimator surface (Fig. 1a and Table 2). The scan duration ranged from 5 to 20 min.

For tomographic acquisitions, cylindrical phantoms with hot spherical and/or cylindrical inserts were used (Fig. 1b and Table 2). The total volume of the hot inserts varied between experiments and ranged from 58 to 560 mL, while the volume of the cylinder was about 6 L (Jaszczak phantom) and 10 L (Elliptical Thorax phantom). In the experiments where inserts were placed in the hot background, the ratio of sphere to background activity concentration was always close to 6:1 (which corresponds to that often observed in clinical studies).

For each phantom configuration and each experiment, the total activity in the phantom was sufficiently low that the camera did not display any dead time effects. For all scans, the projection data were acquired in three abutting energy windows, namely the 20% photopeak window (PW), the lower scatter window (LSW) and the upper scatter window (USW). The data in these three windows were subsequently used to perform triple energy window (TEW) scatter correction. The acquisition times ranged from 8 to 40s per projection with a total of 60–96 projection (30–48 camera stops). Table 3 provides energy window settings used in our experiments and simulations (for ^177^Lu, only the 208 keV photopeak was used).

**Table 3:** Energy window settings for ^131^I [37], ^177^Lu [38], and ^188^Re [39] used in the experimental acquisitions and in the simulations

Isotope	Photopeak window (PW) [keV]		Lower scatter window (LSW) [keV]		Upper scatter window (USW) [keV]	
	Center	Range	Center	Range	Center	Range
^131^I	364	328–400	317	306–328	411	400–422
^177^Lu^a^	208	187–229	167	146–187	249	229–270
^188^Re	155	140–171	136	132–140	175	171–178

^a^For experiments acquired at Quebec (experiment D), the range of LSW and USW were 166–187 and 229–250, respectively

### Monte Carlo simulation experiments

The Geant4 Applications for Tomographic Emission (GATE version 6.1 [27]) Monte Carlo code was used for the simulated experiments. The Siemens SymbiaT dual head SPECT imaging system was modeled. The system geometry (detector, collimator and shielding) used in our simulations was identical to that described and validated in our previous study [28].

The emission energy spectra of the three isotopes, which have complex decay schemes, are built-in into GATE and included accurate modeling of *β*^−^ and gamma emissions. The simulated radionuclides were distributed uniformly within their respective source volume, as described in the next paragraph.

For each radionuclide, four phantom configurations (analogous to those used in the experiments) were simulated:

I. Point source (1-mL sphere) in air
II. 100-mL spherical source placed in the center of a cylinder filled with non-radioactive water
III. 100-mL spherical source placed in the center of a cylinder filled with radioactive water
IV. Cylinder filled with uniform activity

In all simulation experiments, the phantoms were placed at the center of the field of view (FOV) of the camera. The distance from the source to each of the collimator surfaces was equal to 25 cm. The cylindrical phantom used in these simulations had the same dimensions as that used in the experiments. Although multiple inserts with different sizes were used in the phantom experiments, while only a single sphere was used in the simulations, the characteristics of photons recorded by the camera when using this simple phantom model were very similar to those from the experiments, providing us with information sufficient to explain discrepancies in CF values obtained by different methods.

The total number of decays (*N*_tot_) simulated for each phantom configuration and corresponding activities (assuming in each case 5 min acquisition time) are listed in Table 4 (*N*_tot_ was selected so that the total number of photons detected in PW was more than 15,000 in order to ensure errors are < 1%). For each simulation experiment, the projection images corresponding to the true primary photons and the total photons recorded in the photopeak window (PW), as well as those recorded in the two scatter windows (LSW and USW), were generated.

**Table 4:** Total number of decays used in the simulation experiments. Additionally, for each radioisotope, activities (in MBq) corresponding to these simulations, assuming 5-min acquisition times, are provided (in brackets)

	*N*_tot_ (total activity [MBq])					
Isotope	Conf. A ➔ PS	Conf. B ➔ HS + CB	Conf. C^a^ ➔ HS + WB			Conf. D ➔ HC
^131^I	5E8 (1.7)	1E9 (3.3)	Sphere	2.6E8	(0.9)	3E9 (10)
			Bkg	2.7E9	(9)	
^177^Lu	1E9 (3.3)	2E9 (6.7)	Sphere	1.7E9	(5.7)	2E10 (66.7)
			Bkg	1.8E10	(60)	
^188^Re	3.5E8 (1.2)	2E9 (6.7)	Sphere	1.1E9	(3.7)	1.2E10 (40)
			Bkg	1.2E10	(40)	

^a^The number in decays in the sphere and the background was specified so that the ratio of activity concentrations was equal to 6

For all phantom configurations, only one planar projection was simulated for each of the photopeak windows (PW) and for each of the two scatter energy windows (LSW and USW). Benefiting from the cylindrical symmetry of the simulated phantoms, the tomographic images were created by replicating these single projections 90 times with Poisson noise added to the data.

### Image reconstruction

The images from the experimentally acquired tomographic projection datasets, as well as these from simulations, were reconstructed using in-house developed software packages (MIRG software [29] for ^177^Lu and ^188^Re, UM software [30] for ^131^I). In all cases, the OSEM algorithm (see Table 5 for details), with CT-based attenuation correction and TEW scatter correction [31, 32], was employed.

**Table 5:** Parameters used in the reconstructions of images from experimental and simulated tomographic data (experiments performed using configurations HS + CB, HS + HB and HC)

Isotope	Reconstruction	Iterations	Subsets
^131^I	UM Software [30]	35	6
^177^Lu^a^	MIRG qSPECT [29]	6	10
	Siemens Flash3D [20]	6	10
^188^Re	MIRG qSPECT [29]	6	10

^a^For the reconstruction of phantom experiment D (performed at Quebec), 12 subsets which were used as the tomographic data were collected with 96 projections

Additionally, ^177^Lu datasets were reconstructed using the Siemens software available on the camera (Flash3D) [20]. By definition, these reconstructions included resolution recovery (RR) correction. This correction, however, should have no effect on the total number of counts recorded in the reconstructed image. Therefore, CFs obtained from images reconstructed with and without RR should be considered to be equivalent. In all cases, the matrix size was 128 × 128 × 128 with the pixel size equal to 4.79 mm.

Moreover, for each isotope and each phantom configuration, the images were reconstructed from the simulated data corresponding to the primary photons only. In this case, no scatter correction was required so only attenuation correction was included in the reconstruction. The attenuation maps used in all reconstructions of the simulated data were generated using cylindrical phantom shapes filled with narrow-beam attenuation coefficients.

### Determination of camera calibration factor

The camera calibration factor (CF) can be determined using the following general formula:

1 $$ \mathrm{CF}=\frac{C}{A\ t} $$

where *C* is the number of photons emitted by the source having the activity *A* and recorded by the camera in time *t*. This general formula formed the bases of all our data processing; the details of calculations are summarized in Table 6. For simulated data, the product of activity and time was replaced by the total number of decays.

**Table 6:** Techniques used in CF determination from the experimental and simulated data

Config.	CF	Definitions		
		Counts	Time	Activity
Phantom experiments				
A	CF_PWSC_	Count in PW corrected for scatter using TEW: *C*_PWSC_	Scan time: *t*	Small source activity: *A*
B	${\mathrm{CF}}_R^B$	Total counts in the image reconstructed with AC + SC: *C*_*R*_	Number of projections multiplied by the projection duration: *n*_*p*_*t*_*p*_	Total activity in spheres: *A*
C	${\mathrm{CF}}_R^C$			Total phantom activity (spheres+bkg): *A*
D	${\mathrm{CF}}_R^D$			Total activity in phantom: *A*
Simulation experiments				
A	CF_PWSCsim_	Count in PW corrected for scatter using TEW: *C*_PWSCsim_	Total number of simulated decays: *N*_*tot*_	
	CF_PPsim_	Primary photons simulated in PW: C_PPsim_		
B	${\mathrm{CF}}_{R\mathrm{sim}}^B$	Total counts in the image that was reconstructed from PW with AC + SC: *C*_*R*sim_	Number of projections multiplied by number of decays simulated in each projection: *n*_*p*_*N*_*tot*_	
	${\mathrm{CF}}_{R\mathrm{PPsim}}^B$	Total counts in the image reconstructed from primary photons only with AC: *C*_*R*PPsim_		
C	${\mathrm{CF}}_{R\mathrm{sim}}^C$	Total counts in the image that was reconstructed from PW with AC + SC: *C*_*R*sim_		
	${\mathrm{CF}}_{R\mathrm{PPsim}}^C$	Total counts in the image reconstructed from primary photons only with AC: *C*_*R*PPsim_		
D	${\mathrm{CF}}_{R\mathrm{sim}}^D$	Total counts in the image that was reconstructed from PW with AC + SC: *C*_*R*sim_		

#### Planar acquisitions (experimental and simulated configurations—PS)

For planar acquisitions, the CF was directly calculated from the acquired planar images; no reconstruction was required. The counts collected in the entire field of view of the camera were employed and *C*_PWSC_ corresponding to the PW counts corrected for scatter using the TEW method was used.

Additionally, our simulated data provided us with the estimate of the number of primary photons. This allowed us to calculate *C*_PPsim_ (the “true” CF), which was not affected by approximations related to the TEW scatter correction.

#### Tomographic acquisitions (experimental and simulated configurations—HS + CB, HS + HB and HC)

For tomographic phantom experiments, the total numbers of counts, summed over the entire 3D image, were used to determine the CFs corresponding to each isotope and each phantom configuration.

Additionally, for simulated data, the CF factors were calculated using the images reconstructed from primary photons only (see Table 6).

In Table 6, the CF symbols corresponding to the values obtained from planar data are marked with subscript *PW* for “photopeak window” and *PWSC* for “photopeak window scatter corrected”; CF obtained from tomographic data are marked with subscript *R* for “reconstructed” and superscript *B, C or D* indicating configuration of the phantom. Furthermore, the CF obtained from simulated data was labeled with subscript *sim*, while CF calculated from primary photons only are additionally marked with subscript *PP.*

## Results

**Fig. 2:**
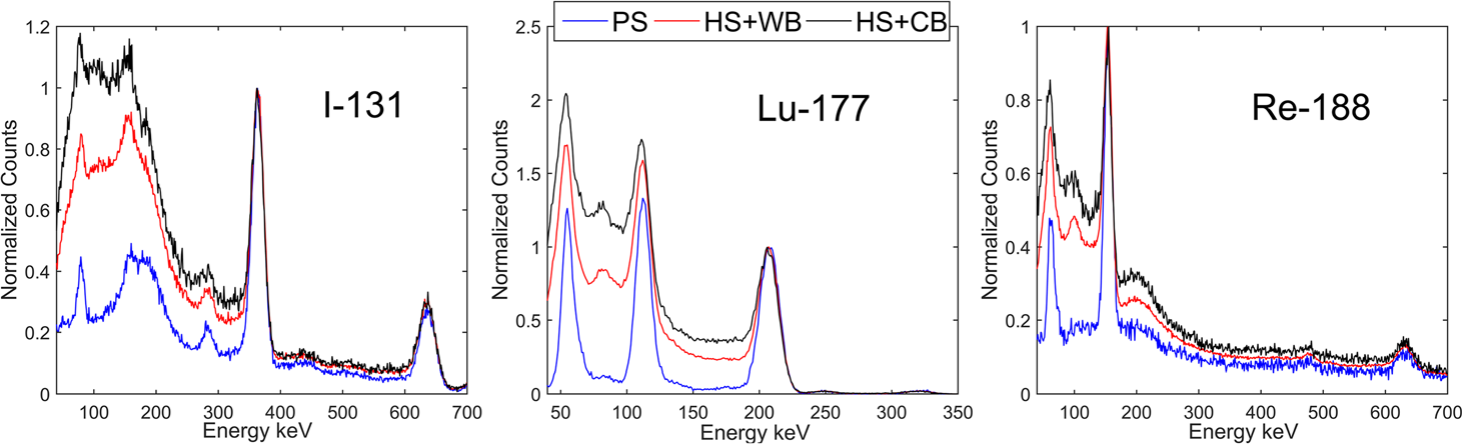
Simulated energy spectra as would be acquired by the SPECT camera from emissions of ^131^I, ^177^Lu, and ^188^Re. For each isotope, a point source scanned in air (blue line) and a 100-mL hot sphere placed at the center of a 20-cm diameter cylindrical phantom filled with non-radioactive water (black line) and warm (red line) water were simulated

Figure 2 presents the energy spectra for the three investigated radioisotopes, generated by our GATE simulations. The phantoms used in these simulations corresponded to a point source scanned in air (blue line), a 100-mL sphere filled with activity placed at the center of a cylinder filled with cold (black line) and warm (red line) water.

**Fig. 3:**
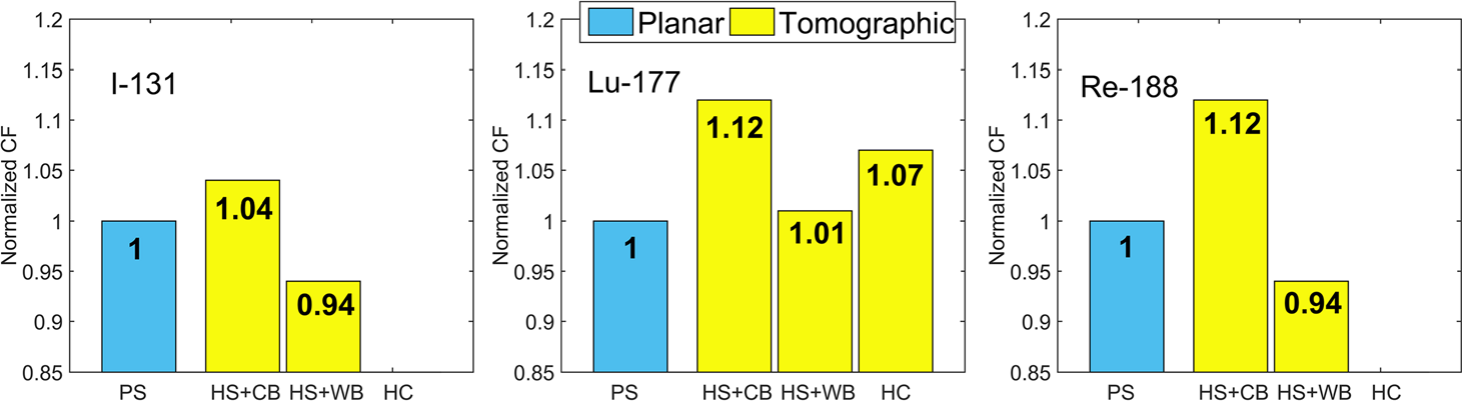
Summary of CF obtained experimentally using different phantom configurations. The data were normalized using counts in the planar acquisition of a point source corrected for scatter with TEW

**Fig. 4:**
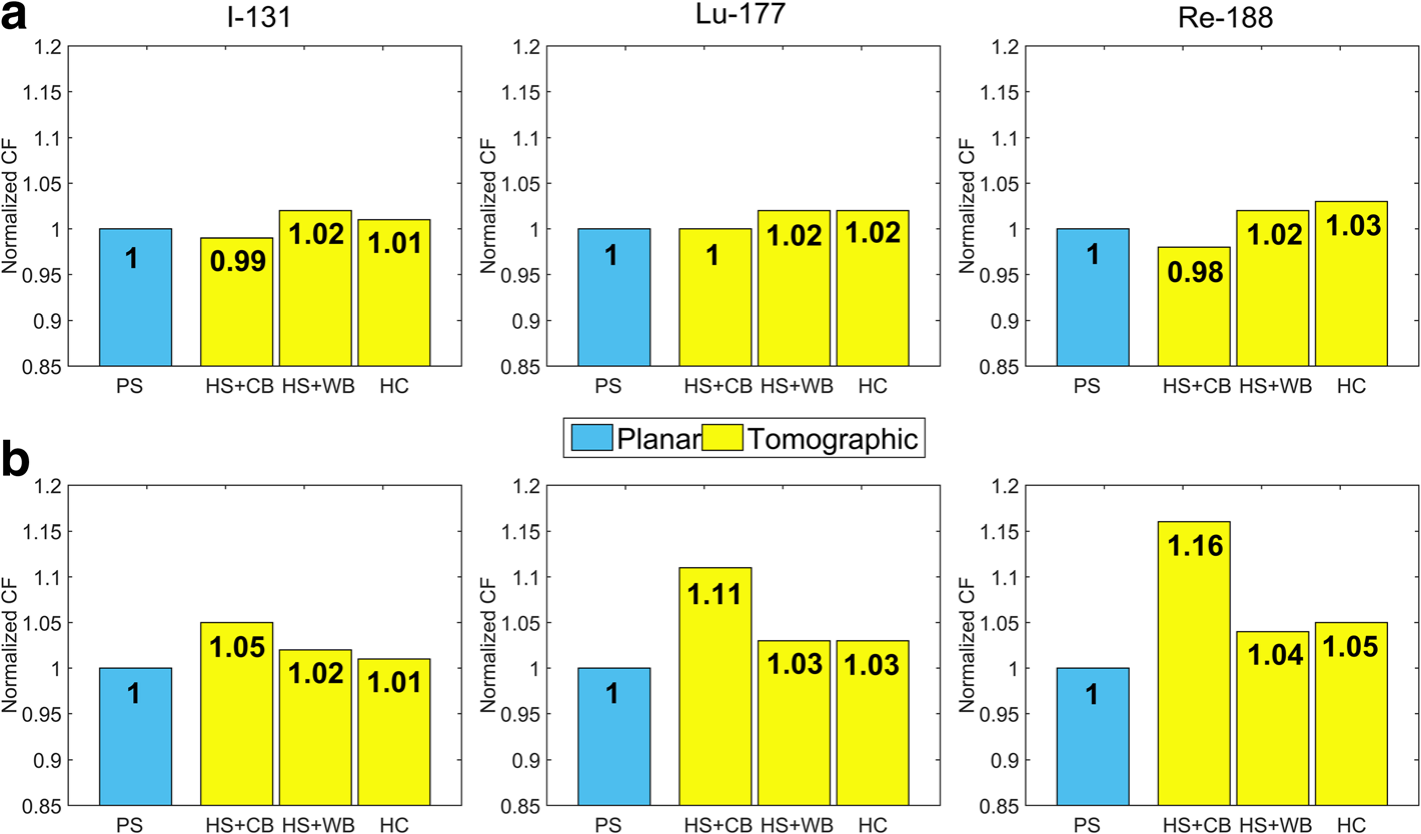
Summary of CF obtained from simulated phantom experiments performed using different phantom configurations. **a** shows CFs obtained from primary photons only normalized using CF_PPsim_, while CFs shown in **b** were calculated using total counts recorded in the photopeak window corrected for scatter with TEW and normalized using CF_PWSCsim_

Tables 7 and 8 summarize the CF values obtained using all planar and tomographic configurations (as outlined in Table 6) from simulations and phantom experiments, respectively. Additionally, these results are presented in a graphical form in Figs. 3 and 4. Since the CF values for ^177^Lu data obtained from MIRG and Siemens reconstructions agreed to within 3%, only CF from MIRG reconstructions were used in the subsequent analysis.

**Table 7:** Experimental camera CF determined using different phantom configurations

Experiment number	Isotope	Experimental configuration	CF [cps/MBq]	Mean CF value [cps/MBq]
1	^131^I	A ➔ PS	58.32	58.3
2		B ➔ HS + CB	59.94	60.5
3			61.10	
4		C ➔ HS + WB	56.91	55.0
5			53.05	
6	^177^Lu	A ➔ PS	9.94	9.4
7			8.93	
8		B ➔ HS + CB	11.04	10.5
9			9.87	
10		C ➔ HS + WB	9.75	9.5
11			9.68	
12			9.84	
13			8.90	
14		D ➔ HC	10.10	10.1
15	^188^Re	A ➔ PS	15.8	16.5
16			17.56	
17			15.99	
18		B ➔ HS + CB	18.64	18.5
19			18.26	
20		C ➔ HS + WB	15.09	15.5
21			15.95

**Table 8:** Camera CF obtained using simulated data

Isotope	Configuration A [cps/MBq]		Configuration B [cps/MBq]		Configuration C [cps/MBq]		Configuration D [cps/MBq]	
	CF_PWSCsim_	CF_PPsim_	${\mathrm{CF}}_{R\mathrm{sim}}^B$	${\mathrm{CF}}_{R\mathrm{PPsim}}^B$	${\mathrm{CF}}_{R\mathrm{sim}}^C$	${\mathrm{CF}}_{R\mathrm{PPsim}}^C$	${\mathrm{CF}}_{R\mathrm{sim}}^D$	${\mathrm{CF}}_{R\mathrm{PPsim}}^D$
^131^I	65.74	66.51	69.23	65.55	67.05	67.63	66.54	67.04
^177^Lu	11.18	11.33	12.44	11.32	11.49	11.59	11.51	11.54
^188^Re	17.60	18.37	20.47	17.98	18.29	18.77	18.51	18.98

In order to facilitate comparison of CFs obtained from different experiments with different phantom configurations, the CF values in Figs. 3 and 4 are presented in relative units. For simulated data, shown in Fig. 4a, CF obtained from primary photons recorded in the photopeak window of the planar acquisition of a point source were considered to be the “true” CF values and were set to 1. For the experimental data presented in Fig. 3 and for simulations shown in Fig. 4b, the data were normalized using counts in the planar acquisition of a point source corrected for scatter, i.e. CF_PWSC_ and CF_PWSCsim_, respectively.

## Discussion

The spectra presented in Fig. 2 allow us to evaluate the contribution of scattered photons to the photopeak energy window for different phantom configurations. While scatter component in point source (PS) scans of ^177^Lu is relatively low, for ^131^I and ^188^Re, the photons from high-energy gamma transitions, which were scattered mostly in the camera and its components, substantially increase the background. This observation supports our claim that scatter correction should be performed when CF is derived from the data obtained using planar scans of point sources. The scatter correction method, which is the most popular in clinics, is TEW. Besides being simple and easy to implement, TEW allows us to correct not only for self-scattered photons, but also for high-energy scatter and other background.

Further analysis of the data presented in Fig. 2 confirms that scatter correction should be included in all tomographic image reconstructions. All energy spectra for HS + CB and HS + WB phantom configurations that were used in our tomographic acquisitions, and which model patient scans better than point sources, display large scatter background under the photopeaks.

For all isotopes (^131^I, ^177^Lu and ^188^Re), the experimental CFs (summarized in Fig. 3 and Table 7) show relatively good agreement between CF_PWSC_ obtained from planar scans corrected for scatter and ${\mathrm{CF}}_R^C$ obtained from tomographic scans performed using hot sources placed in warm background (HS + WB). These CF values agree to within 6%. The agreement usually improves (to below 3%) when CF obtained from the experiments performed on the same day are considered. This improvement may be attributed to the fact that for the same-day experiments, all errors in activity determination are minimized, as the activity measurements are performed using the same vial and same dose calibrator settings. However, the differences between CF_PWSC_ and ${\mathrm{CF}}_R^B$ (HS + CB) values are much larger, for ^177^Lu and ^188^Re even reaching 12%.

The explanation of all these effects can be provided by the analysis of our MC simulation results. Firstly, as expected, when considering only primary photons, for all radioisotopes, CFs obtained from planar scans (CF_PPsim_) and those reconstructed from tomographic data (with attenuation correction) agree to within 1–3% (see Table 8 and Fig. 4a). Such small differences may be caused by statistical fluctuations and small approximations in attenuation correction used in reconstructions of simulated tomographic data (voxelized attenuation maps were used in reconstructions, while in GATE analytical shapes are used).

**Fig. 5:**
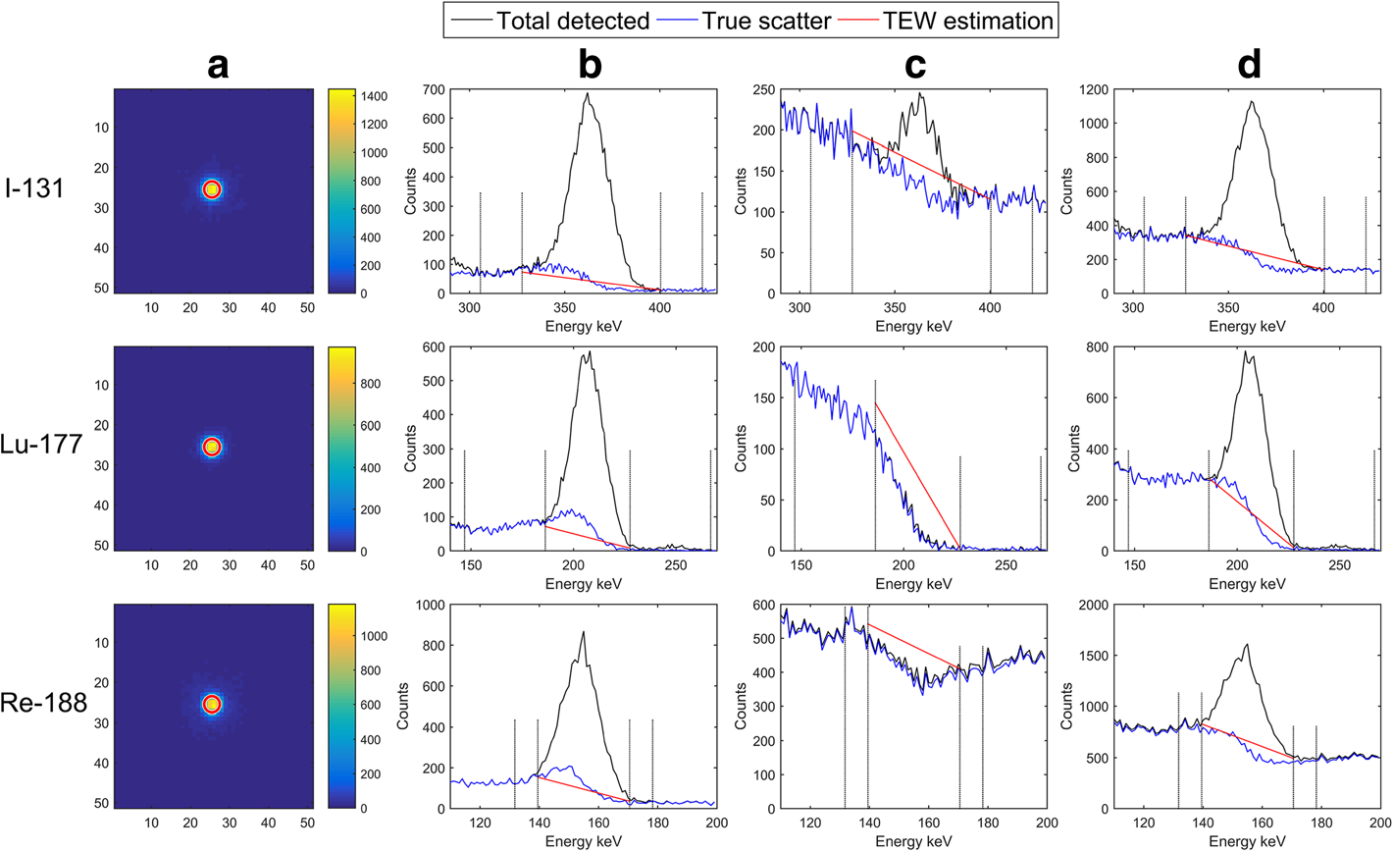
The energy spectra obtained from the simulations of the phantom scanned in configuration B (HS + CB). The counts recorded in the photopeak window and correspond to the ROI drawn on the projection images: around the hot object (column **b**), in the background surrounding this ROI (column **c**), and in the entire image (column **d**). Column **a** shows the simulated PW projections. The hot object ROI was placed inside the red circle while the background ROI comprised all counts found on the outside of the red circle

However, larger discrepancies, similar to those observed in experimental data, are found when comparing CFs obtained from simulated PS scans corrected for scatter CF_PWSCsim_ and simulated tomographic scans (Table 8 and Fig. 4b). The differences between CF_PWSCsim_ and both ${\mathrm{CF}}_{R\mathrm{sim}}^C$ and ${\mathrm{CF}}_{R\mathrm{sim}}^D$ remain below 5%, while ${\mathrm{CF}}_{R\mathrm{sim}}^C$ and ${\mathrm{CF}}_{R\mathrm{sim}}^D$ agree with each other to within 1%. However, the differences between CF_PWSCsim_ and ${\mathrm{CF}}_{R\mathrm{sim}}^B$ increase to 12–16%. These effects are caused by the approximations of the TEW scatter correction method, which can be visualized when considering the shapes of the profiles presented in Fig. 5.

Simulated spectra presented in Fig. 5 correspond to the phantom configuration B (HS + CB). The graphs compare the shapes of the photopeak, the true scatter component observed in PW and the scatter estimated by the TEW method using counts recorded in LSW + USW. Counts in the three regions were analyzed. Spectra presented in Fig. 5b correspond to counts recorded in the source ROI, and Fig. 5a shows the location of the source ROI drawn in the projection images of each isotope. Spectra in Fig. 5c correspond to counts recorded in the background region around the source, and Fig. 5d shows spectra of counts recorded in the entire image (these counts were used for the CF determination). Please note that Fig. 5c for ^131^I contains a small peak corresponding to septal penetration of the collimator by 364 keV photons.

The analysis of these graphs clearly demonstrates that for all isotopes, the TEW method (area under the red line) underestimates the true scatter (marked by the blue line) in the source ROI region while overestimates it in the background region. As a result, the source region seems to have more counts, while counts in the background around the source seem to be lower than they should be. This “surplus” is further enhanced by the attenuation correction, which boosts the excess of counts in the source region, because it is located in the center of the phantom where attenuation correction is the highest.

On the other hand, when scatter correction is done by subtracting projections, before image reconstruction, the overestimation of scatter counts in the background may potentially create negative counts. However, in our reconstructions, these negative counts could not occur because the TEW scatter estimate was included in the denominator of the OSEM formula. As a result, the total number of reconstructed counts used for CF calculation is higher than the truth and also higher than that determined from planar scans. This effect is relatively smaller for phantom configurations in which activity is distributed over the entire phantom (HS + WB and HC).

Additionally, please note that although CFs determined experimentally and obtained from simulations are quite similar, CF from simulations exceed experimental values by 3–10%. In our opinion, these differences should be attributed to approximations made in the simulated camera model [28] and inaccuracies in dose calibrator measurements of source activities.

At this point, it is important to emphasize that the CF value, as defined in our study, corresponds purely to the camera efficiency for given radioisotope, collimator and energy window settings. It does not depend on the camera and image resolution, the size and shape of the imaged object, the signal-to-background ratio and other factors. Although some authors propose to combine CF and RC into a single calibration coefficient [33], such approach would be very challenging, as it is impossible to design a calibration experiment which would model every patient geometry and every activity distribution. More importantly, in order to account for these different conditions, such a “combined” approach would require not a single value of CF, but a large table of values, which additionally would depend on the segmentation method that was used to generate RC.

This is not to say that the proposed CF allows us to avoid the challenges related to image segmentation. Still, the activity maps, which are obtained by multiplying patient images (i.e. count-maps) by CF, must be segmented if one wants to get activity in any particular volume. The advantage of the proposed method is that CF determined using a single planar scan can be repeatedly applied to many patient studies, as long as they were acquired using the same camera, collimator, radioisotope and energy window settings. It has been shown that, under normal exploitation conditions, the camera sensitivity (thus this CF) will remain unchanged over a long period of time [18].

Actually, another observation from this study (and also from our previous experience) is that often calibration experiments performed using the same type of camera (from the same manufacturer) and same acquisition protocols (collimator and energy window settings) but located in different Nuclear Medicine departments (often even in different countries) result in very similar values of CFs. This fact may be illustrated by the experimental CFs for ^177^Lu phantom configuration C and D, which agree well (within 6%) in spite of the fact that one of these studies was done using Siemens camera located in Vancouver and the other camera in Quebec City.

## Conclusions

Accurate determination of the gamma camera CF is critical for quantitative imaging to translate counts in the reconstructed images into activity values. However, currently, there is no consensus about the calibration method, both planar and tomographic scans have been performed and the resulting CF applied in research and clinical studies.

We have shown that planar acquisition of a point-like source provides CF very close to those obtained from tomographic images (reconstructed with attenuation and scatter corrections) of a phantom where the activity is distributed over the entire volume (with or without the hot object(s) in its center). The value of CF determined using these two approaches agree to within 3% when experiments are performed on the same day and to 6% for experiments done over the period of several months. Usually, such phantom configuration is considered to be a good approximation of activity distribution encountered in clinical patient studies. However, our analysis suggests that, for all investigated radiotherapy isotopes, the camera calibration based on a planar scan of a point source must include scatter correction. This is because photopeak windows for ^131^I and ^188^Re, and to a lesser degree for ^177^Lu, contain important component from scattered high-energy gamma emissions (and septal penetration for ^131^I).

Additionally, our experiments indicate that camera calibration performed using tomographic scan of a source(s) placed in non-radioactive (cold) background may overestimate CF by more than 10%. Thus, the use of this method is not recommended for determination of the camera CF. Analysis of simulations helped us to understand that this rather large discrepancy is due to approximations made by the TEW scatter correction, even further enhanced by attenuation correction performed during image reconstruction.

Based on these considerations, we conclude that camera CF may be confidently determined using planar scans of the point source, provided that the background contribution to the photopeak is removed, for example using the TEW method. The approach is simple and easy to perform and provides CF with sufficient accuracy (~ 5%) to be used in clinical quantitative imaging studies. The proposed method is general and is expected to provide good results for other isotopes than those reported here.

## Abbreviations

3D: Three-dimensional
CB: Cold background
CF: Calibration factor
FOV: Field of view
GATE: Geant4 Applications for Tomographic Emission
HC: Hot cylinder
HS: Hot sources
LSW: Lower scatter window
PP: Primary photons
PS: Point source
PVE: Partial volume effects
PW: Photopeak window
RC: Recovery coefficients
ROI: Region of interest
RR: Resolution recovery
SPECT: Single photon emission computed tomography
TEW: Triple energy window
TRT: Targeted radionuclide therapies
USW: Upper scatter window
WB: Warm background
